# A Decision-Theoretic Perspective on Fairness in Clinical Predictive Models

**DOI:** 10.21203/rs.3.rs-9644545/v1

**Published:** 2026-05-08

**Authors:** Joshua W. Anderson, Nader Shaikh, Gregory F. Cooper, Shyam Visweswaran

**Affiliations:** 1 Intelligent Systems Program, University of Pittsburgh, PA, USA; 2 Department of Pediatrics, University of Pittsburgh, PA, USA; 3 Department of Biomedical Informatics, University of Pittsburgh, PA, USA

## Abstract

Fairness is an important concern in statistical models, especially in clinical prediction models. Most fairness methods focus on model predictions, aiming for parity in model performance across relevant groups. However, this approach overlooks the broader implications of fairness when these models are used in clinical decision-making. We argue that prediction-based fairness frameworks, while valuable, are inherently limited when patient outcomes are equally, if not more, important concerning fairness. We analyze a deployed clinical prediction model, UTICalc, which was revised to improve fairness across racial groups and showed improved performance on a prediction-based fairness metric, namely, equal opportunity (equal true positive rate). We developed a decision-theoretic framework to assess the fairness of UTICalc by integrating patient outcome utilities with model predictions. To this end, we constructed a decision tree to model the clinical decision-making process for assessing and treating urinary tract infection (UTI) in young children, for which UTICalc was developed. Our results show that the revised UTICalc model did not improve an outcome-based fairness metric, namely, expected utility parity. This suggests that prediction-based and outcome-based fairness may diverge, with implications for clinical settings. Furthermore, we suggest that fairness in clinical prediction models should be evaluated based on patient outcomes as well as model predictions.

## Introduction

Clinical prediction models estimate the risk of a disease or outcome from an individual’s data and are routinely used for risk assessment, diagnosis, treatment decisions, and resource allocation to improve health outcomes^1^. Two common types of models are diagnostic prediction models, which estimate an individual’s probability that a particular disease is currently present, and prognostic prediction models, which estimate an individual’s probability of developing a disease in the future. These models range from statistical models that use a few variables as input to artificial intelligence/machine learning (AI/ML)-based models that use hundreds of variables. However, many models are not extensively evaluated, and there is concern, especially with data-driven AI/ML models, about the potential to introduce or amplify health inequalities. Biased models can lead to inequalities in clinical decision-making, differential treatment recommendations, and ultimately, unequal health outcomes, especially for minority populations^2^. Hence, there is a need to assess and improve the fairness of biased models used in healthcare.

Membership in minority groups may be represented in prediction models by including attributes such as race, sex, and age as predictor variables. The inclusion of race as a predictor variable in deployed clinical prediction models has come under increasing scrutiny, given mounting evidence that their use may introduce or exacerbate health inequalities across racial groups. When race is included as a proxy for genetic or biological differences, it risks misrepresenting the intended information by embedding social prejudices or stereotypes rather than meaningful biological variation. Such models have been shown to be racially discriminatory by unfairly assigning risk to Black, Hispanic, and other minority groups, leading to reduced or inappropriate care^3,4^. Yet, these examples do not fully resolve questions about the benefits or harms of including race as a predictor in clinical models. The consequences of including or excluding race are not straightforward: removing race from a model may reduce one form of bias while introducing others, and the net effect on patient outcomes depends heavily on the clinical context in which the model is deployed. This ongoing uncertainty has motivated a growing body of research on fairness in clinical models that incorporate race or similar attributes.

Model fairness in healthcare is a fast-growing field of study focused on measuring fairness and developing fair prediction models optimized to “ensure equality in patient outcomes, performance, and resource allocation^5^.” A wide range of metrics has been developed to assess fairness in clinical models and to optimize model performance, ensuring equality in outcomes across racial groups^6^. These metrics directly measure model predictions (such as disease probabilities or risk categories) and evaluate differences in accuracy, sensitivity, specificity, and predictive values across predefined racial groups. Such prediction-based fairness metrics have become the dominant framework for evaluating model fairness in healthcare^7^. Furthermore, computational methods have been developed to improve fairness by mitigating bias at various stages of model development: pre-processing to remove biases from the data before model training, in-processing to explicitly train models with fairness constraints, and post-processing to correct biased model predictions^8^.

However, a limitation of current fairness metrics is that they evaluate only the statistical properties of model predictions, not the clinical consequences of the decisions those predictions inform. In clinical practice, the guidance from a prediction model is only one of the factors a clinician considers when making decisions for a patient, alongside items such as patient preferences and costs. Thus, the patient’s outcome, which depends in part on the clinician’s decision, is not determined solely by the prediction model, especially considering that clinicians will not always adhere to model recommendations. Equalizing predictive performance across racial groups does not guarantee equal decisions, and equal decisions do not guarantee equal outcomes. Evaluating fairness solely through predictive metrics, therefore, provides an incomplete picture of whether a model produces fair outcomes across racial groups.

Recent work has begun to investigate how racial bias affects clinical prediction models in terms of model predictions and patient outcomes. Coots et al.^9^ develop a decision-theoretic framework comparing race-aware and race-unaware models, evaluating the effect of race across three dimensions: model calibration, clinical decisions, and outcomes measured by net benefit, a metric that quantifies the difference between expected gains and costs but assumes linear additivity. Building on this, Benitez-Aurioles et al.^10^ derive a net-benefit function for model-backed decision policies expressed as a weighted sum of true positives (benefit) and false positives (cost). While these works establish a foundation for decision-theoretic analysis of clinical prediction models, both rely on net benefit, which, though practical and interpretable, is a function of model predictions and does not account for nonlinear preferences such as risk aversion as is possible under a decision-theoretic framework. Furthermore, the decision problems are framed as standard gambles rather than fully specified decision trees, which limits their applicability to the complexities of real clinical settings.

In this article, we developed a decision-analytic framework that combines model predictions with the utilities of patient outcomes, offering a more clinically grounded approach to fairness evaluation^9–11^. We applied this framework to Urinary Tract Infection Calculator (UTICalc), a clinical decision-support tool that uses prediction models to estimate the risk of urinary tract infection (UTI) in young febrile children^12,13^. UTIs are among the most common serious bacterial infections in young children, affecting approximately 7% of febrile children under 2 years of age who come to emergency departments^14^. The diagnosis of UTI requires a urine sample for analysis, and since children this young are unable to provide a sample on demand, obtaining it without delay requires urinary catheterization, which is an uncomfortable procedure. An accurate estimate of the probability of UTI based on the child’s clinical information can guide whether catheterization is needed. UTICalc was developed as a diagnostic prediction model from data obtained from electronic health records (EHRs). The first version of UTICalc (UTICalc V1), developed in 2018, is a logistic regression (LR) model for predicting UTI risk in febrile children aged 2 to 23 months, using 5 binary clinical predictors: age (<12 months vs. >=12 months), sex (female or uncircumcised male vs. circumcised male), race (Black vs. nonblack), fever magnitude (>39°C vs. =<39°C), and alternate fever source (absence of another source of fever vs. presence). A probability of 2% or higher is the cutoff for recommending a catheterized urine sample, and at this threshold, the model achieved a sensitivity of approximately 95% and specificity of 35% for correctly predicting a UTI. UTICalc V1 also included a second LR model that used urinalysis (UA) results as predictors: nitrites (yes vs. no), leukocyte esterase (trace, small, moderate, large), white blood count per cubic millimeter (value), and bacteria on Gram stain (yes vs. no), in addition to the clinical predictors, to estimate UTI risk. A probability of 5% or higher is the cutoff for recommending antibiotic treatment, and at this threshold, the model achieved a sensitivity of 96% and a specificity of 93%^12,13^. We refer to the first LR model the clinical model and the second LR model as the UA model.

In 2020, UTICalc Version 2 (UTICalc V2) was developed to reexamine the effect of race as a predictor^13^. Analyses showed that removing race narrowed the gap in true positive rates across racial groups but had a negligible impact on the overall number of UTIs diagnosed. Thus, the deployed clinical model for UTICalc V2 was identical to UTICalc V1, with the addition of an alert flagging patients in which race was the determining factor for a positive prediction. Because V2 was subsequently superseded by later updates, it is not included in our analyses.

In 2022, UTICalc Version 3 (UTICalc V3) was developed to remove race as a predictor and to instead include two predictors, history of UTI and duration of fever, due to concerns about racial bias. Thus, UTICalc V3 has a clinical model developed from data from the same cohort using 6 binary clinical predictors: age (<12 months vs. >=12 months), sex (female or uncircumcised male vs. circumcised male), fever magnitude (>39°C vs. =<39°C), alternate fever source (absence of another source of fever vs. presence), duration of fever (>=48 hours vs. <48 hours), and history of UTI (yes vs. no). A probability of 2% or higher is the cutoff for recommending a catheterized urine sample, and at this threshold, the model achieved a sensitivity of approximately 96% and specificity of approximately 35%. The UA model, which used UA results as additional predictors, had 92% sensitivity and 94% specificity at the 5% risk threshold^12,14^.

In practice, UTICalc (V1 and V3) operates in two stages. First, the clinical model is used to compute a prior probability of UTI based on clinical predictors alone, without UA results. If this probability exceeds 2%, catheterization is performed to obtain a urine sample for UA. Second, the UA model (V1 and V3) incorporates both clinical predictors and UA results to estimate a posterior probability. If this probability exceeds 5%, antibiotics are initiated. Table 4 defines each model and specifies what probability it estimates.

We evaluated the fairness of two versions of UTICalc clinical models (only the first LR model), one including race as a predictor and one excluding it (V1 and V3, respectively), across Black and nonblack groups using a standard performance–based fairness metric and compared these results with our decision-analytic approach. In the decision-analytic approach, we evaluated the fairness of two versions (V1 and V3) of UTICalc, including both the clinical and UA models. We propose that integrating decision analysis into fairness evaluation is a step toward ensuring that clinical prediction models are assessed not only on their predictive performance but also on how they inform decisions that ultimately affect patient outcomes.

## Results

### Prediction–Based Fairness

To compare prediction-based fairness between the clinical models V1 and V3, we used equal opportunity as the fairness metric. This metric requires a prediction model to have the same true positive rate (sensitivity) across groups, meaning that children who truly have a UTI are equally likely to be correctly identified regardless of race. Because UTICalc is a screening test, it was designed to have high sensitivity (95% or higher). Therefore, evaluating sensitivity across Black and nonblack children using equal opportunity aligns the fairness assessment with the clinical objective of screening.

Table 1 reports the performance of the clinical model versions with respect to equal opportunity, which is reported as the mean ± standard deviation based on bootstrap resampling, along with the difference between V1 and V3 and the associated p-value testing the null hypothesis that the difference in equal opportunity from V1 to V3 equals 0. V3 performs statistically significantly better than V1 (p < 0.0001), supporting the claim that the revised model achieved greater fairness in predictive performance and that removing race as a predictor resulted in a fairer model.

### Decision Tree

To apply a decision-analytic framework, we constructed a decision tree to represent clinical decision-making for diagnosing and treating a young child with a UTI. Briefly, a decision tree in decision analysis is a graphical model that represents a sequence of decisions, uncertain events, and outcomes. It contains decision nodes, where branches represent the available actions under the control of the decision-maker; chance nodes, where branches represent possible outcomes with specified probabilities; and outcome nodes, which represent the outcomes of each pathway and are assigned values such as utilities or costs. By combining decisions, probabilities, and outcomes, the tree enables calculation of expected utility to identify the action that provides the greatest anticipated benefit.

**Figure 1.**
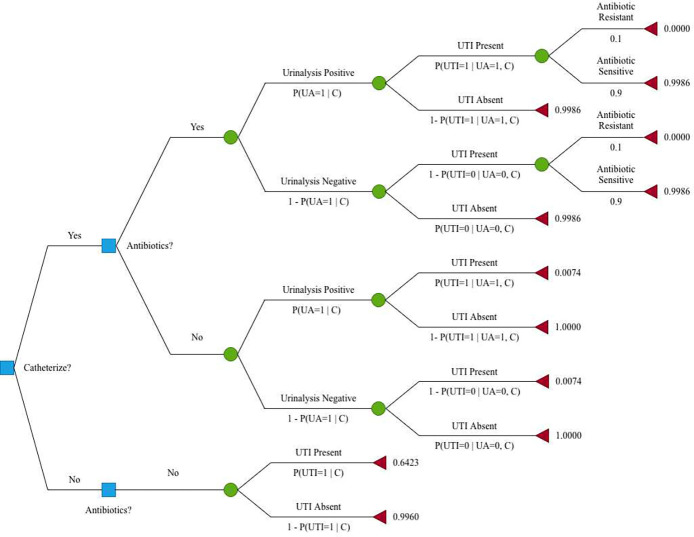
Decision tree used for evaluating fairness. Three types of nodes are present in the tree: decision nodes (blue squares) and associated decision branches, chance nodes (green circles) and associated probabilities of possible events, and outcome nodes (red triangles) with associated costs. UTI denotes urinary tract infection, UA denotes urinalysis, and C denotes patient EHR.

The UTI decision tree is shown in Figure 1. To the left are two decision nodes representing two clinical decisions: whether to perform urinary catheterization to obtain a urine sample and whether to initiate antibiotics. To the right are three types of chance nodes (left to right) representing the probabilities that UA results are positive, that the child is eventually confirmed to have a UTI, and that the infection is resistant to antibiotics. In the case that no catheterization is performed and no antibiotics are given, there is a chance node representing the probability that the patient has a persistent UTI. On the far right are outcome nodes, each with an associated utility determined by the events along the path from the first decision node on the left to the terminal outcome node on the right. The probabilities assigned to the branches of the chance nodes are as follows: for the branches of the UA chance nodes, the UA model outputs the probability of the results being positive in a particular child; for the branches of the UTI chance node, a positive predictive value (PPV) or negative predictive value (NPV) is derived; and for the branches of the antibiotic resistant chance node, we assume a constant probability of 0.1 that the patient is resistant to antibiotic treatment^16^. For the UTI chance node when there is no catheterization or antibiotics, the predicted probability from a given UTICalc version is used. The utilities associated with the outcome nodes are a function of the realistic dollar amounts that an insurer or patient may pay for the events along the path from the first decision node on the left to the terminal outcome node on the right. Table 4 in the Methods section describes each probability term for chance nodes in the tree. Details of the decision tree nodes, estimation of the costs, and derivation of the utilities are provided in the Methods section.

### Decision Analysis-Based Fairness

To compare the fairness between V1 and V3 of both clinical and UA models using the decision tree in Figure 1, we used expected utility (EU) and EU parity. Both the clinical and UA models are necessary to solve the tree, and we apply the same version (V1 or V3) to each LR model. EU is the weighted average of the possible outcomes following a set of decisions, where each outcome is weighted by its probability. EU parity is a measure of differences in the EU across racial groups. In decision analysis, well-calibrated probabilities are essential for obtaining meaningful EU estimates^11^. We assessed calibration using expected calibration error (ECE) and ECE parity (see Supplementary Table 3).

The results presented in Table 2 summarize the performance of V1 and V3 for both clinical and UA models on EU and EU parity. Each metric is reported as the mean ± standard deviation, estimated via bootstrap resampling, along with the difference between V1 and V3 and the associated p-value testing the null hypothesis that the difference in each metric from V1 to V3 equals 0.

For EU, UTICalc V1 and V3 achieve values of 0.9504 ± 0.0042 and 0.9499 ± 0.0040, respectively, with a negligible difference of −0.0005 ± 0.0013, which is not statistically significant at the 0.05 threshold. After recalibration, these values are very similar, with the difference narrowing slightly to −0.0004 ± 0.0037, which is not statistically significant. In terms of EU parity, which measures the difference in utility between racial groups, using the decision tree with UTICalc V1, shows a moderate disparity (−0.0151 ± 0.0066), which improves when V3 is substituted (−0.0068 ± 0.0070), resulting in a positive difference of 0.0083 ± 0.0027. However, this difference is not statistically significant (p-value of 0.4370).

**Figure 2.**
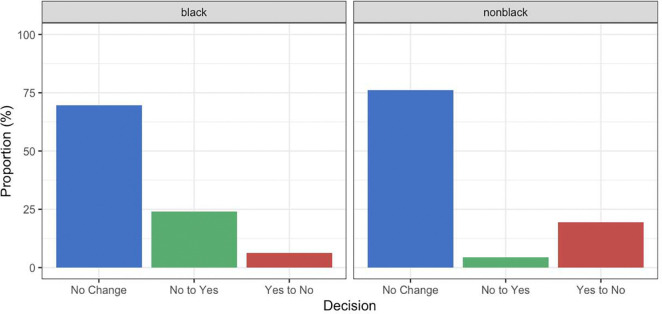
Histograms showing in-group proportion of changes in catheterization recommendations when switching from model versions V1 to V3 in the decision tree in Figure 1. The left panel shows results for Black children, and the right panel for nonblack children. “No Change” indicates that the decision does not change across the models (blue). “No to Yes” indicates that V1 recommended no catheterization, and V3 recommended catheterization (green). “Yes to No” indicates that V1 recommended catheterization and V3 did not (red).

We found no p-values near a level of statistical significance for the change in EU for children. However, we found changed catheterization decisions for many children when moving from V1 to V3, despite the overall catheterization rate remaining similar (see Figure 2).

### Changes in Catheterization Recommendations

Table 1 shows that the overall catheterization recommendation rate in the decision tree is similar across the two model versions, and the difference is not statistically significant at the 0.05 level (p = 0.6490). Figure 2 shows changes in catheterization recommendations across racial groups when switching from V1 to V3 in the decision tree. Among Black children, there were 27 “No to Yes” changes, indicating that V3 resulted in several more catheterizations, compared with only 7 “Yes to No” reversals and 78 unchanged decisions. This suggests that the V1 of the models showed a more conservative strategy for Black patients, reducing catheterization recommendations.

For nonblack children, there were 8 “No to Yes” changes, 35 “Yes to No” changes, and a large majority of 137 unchanged decisions, indicating more stable decision-making compared to Black children. Despite large swings in catheterizations across racial groups, the final catheterization rate was not significantly different, and more importantly, these changes in treatment did not significantly change patient outcomes in either EU or EU parity.

The results show a divergence between prediction-based fairness and outcome-based fairness. Removing race from the UTICalc model significantly improved prediction-based fairness as measured by equal opportunity. However, the expected utility of clinical decisions remained nearly identical across model versions. In other words, improvements in predictive fairness did not translate into improvements in fairness in patient outcomes.

## Discussion

Both UTICalc V1 and V3 performed similarly on EU parity despite statistically significant differences in equal opportunity, reflecting that the models provide comparable clinical utility overall, regardless of whether race is included as a predictor. This supports that prediction-based fairness metrics can yield conclusions that differ from those based on the utility of patient outcomes. The results illustrate the importance of aligning fairness evaluation with the key goal of healthcare: improving patient outcomes.

To construct the decision tree, we make several simplifying assumptions about the clinical decision process. In practice, the decisions made using UTICalc do not strictly follow a decision-theoretic approach, and we constructed a decision tree that is as representative of true clinical decision-making as possible, rather than one that is theoretically ideal. We also made assumptions to specify the utilities. We assumed patients are not re-evaluated recursively upon readmission, treating readmission following antibiotic treatment without further branching. While these assumptions introduce limitations, they reflect the practical constraints of modeling real clinical workflows.

The divergence between prediction-based and outcome-based fairness metrics reflects a broader and more fundamental tension in how fairness is defined in clinical prediction models. Prediction-based metrics, such as equal opportunity, ensure that models are equally predictive across groups, which is a property considered essential for trust, transparency, and interpretability in the algorithmic fairness literature. However, these metrics evaluate model predictions in isolation and do not account for the clinical context in which the predictions are used, the decisions they influence, or the consequences of those decisions for patients. A fairness intervention that improves parity in model predictions may not necessarily improve parity in patient outcomes. We consider this issue to be a fundamental limitation of current fairness evaluation practices for clinical predictive models: that the metrics most used to assess fairness do not measure the outcomes that fairness interventions are ultimately intended to improve.

This study addresses this limitation by providing a framework for measuring fairness that includes outcomes and predictions. By combining probabilities with utilities, decision analysis allows fairness to be assessed in terms of expected outcomes of decisions rather than the statistical properties of model predictions alone. Under this framework, a model that achieves predictive parity may still be considered biased if the consequences of downstream decisions differ across groups. Conversely, differences in predictive performance may be acceptable if they lead to similar or improved outcomes across groups, as in the case of UTICalc.

The implications of this framework extend beyond the specific case of UTICalc. Divergence between prediction-based and outcome-based fairness within a single model suggests that such discordance is inherently possible in any clinical setting where the relationship between predictive performance and outcome utility is not uniform across groups. Incorporating the quality of patient outcomes into fairness evaluations is not always straightforward, as utility estimates may be difficult to measure, context-dependent, or subject to disagreement among stakeholders. Regardless, prediction-based fairness metrics, while valuable for characterizing disparities in model performance, should not be interpreted as comprehensive measures of fairness without also considering the downstream consequences of decisions those models inform.

Aligning fairness goals with the clinical context in which models are deployed requires reconceptualizing what fairness means in practice. Achieving parity in risk prediction may not be sufficient if the utility of patient outcomes is also not considered, since a model that appears equitable under conventional evaluation may still perpetuate or exacerbate disparities in the care patients ultimately receive. Future work should integrate decision-analytic frameworks into both fairness evaluation and model development to properly represent downstream effects of decisions. Doing so would ensure that assessments of equity are grounded in the outcomes that matter most: the health and welfare of the patients these models are designed to serve.

## Methods

### UTICalc Database

The UTICalc database includes training and test datasets extracted from EHRs for 1,686 and 384 children, respectively There are a total of 7 predictor variables in the full dataset, of which 5 are used in V1 and 6 are used in V3. One outcome variable was measured: the presence of a UTI (yes/no), determined by urine culture results. Children were excluded at different rates for model training for UTICalc V1 and V3 due to missing values. In UTICalc V1, data from 1,593 children (1,168 nonblack and 407 Black) were used for training, and in UTICalc V3, data from 1,552 children (1,154 nonblack and 398 Black) were used for training. Additionally, 92 cases were excluded from the test set due to missing values (39 nonblack, 21 Black, and 32 unknown). Approval and waived informed consent were given by the University of Pittsburgh Institutional Review Board for this study. A table summarizing the training and test datasets is provided in Supplementary Table 1.

### Development of a Decision Tree

We developed a decision tree that models the clinical decision-making process in practice. We make a series of assumptions to construct the tree shown in Figure 1, which we believe captures well both real-world clinical practice and near-optimal decision-making as defined by decision theory. The model includes two key decisions: whether to perform catheterization and whether to initiate antibiotics.

The chance nodes in the tree use a variety of probabilistic models to compute probabilities: the two LR models, UTICalc and UA models, and several statistical derivations. Descriptions of the chance node probabilities are given in Table 4. When making estimates with and without race, V1 and V3 are used for both the UTICalc and UA LR models, respectively. Although UTICalc was originally designed to support the catheterization decision, we include the antibiotics decision because the utility of obtaining a urine sample via catheterization depends on how informative it is for guiding effective treatment and the final patient outcome. If catheterization is not performed and antibiotics are not initiated, a chance node represents the probability of a persisting UTI after decisions are made. Since in this case no information would be gained from the decisions made, this probability is simply the predicted probability from UTICalc. If catheterization is performed, a UA test is done immediately on the sample. The probability that the UA will yield a positive or negative result is represented by the chance nodes labeled “Urinalysis Pos/Neg.” Since a decision regarding catheterization must be made before obtaining UA results, it is necessary to estimate the UA results prior to decision-making. The sensitivity of the UA test is known, but individualized probabilities were derived for patients in the UTICalc database. More specifically, the overall probability for the UA being positive can be represented in the tree as:

P(UA=1)=P(UA=1∣UTI=1)⋅P(UTI=1)+P(UA=1∣UTI=0)⋅P(UTI=0)

where P(UA=1) represents the marginal probability of the UA test and P(UTI=1) is the marginal probability of UTI.

Since UA results are available for some patients in our database, we can use this data to estimate the probability of a positive UA, P(UA=1|L), where L represents the UA results. This describes the UA model mentioned in the introduction^12^. Unfortunately, UA results are not known before the decision-maker has made the catheterization decision; therefore, variables L are hidden at the time of decision-making. This means we will not be able to observe L for any given patient when deciding whether to catheterize, even though we have this information for past patients in the training data. To design a model that can be used at the time of decision-making, we must derive a UA model that only depends on known clinical variables C rather than L. We derive a new UA model P(UA=1|C) which can be obtained by marginalizing over L:

$$P(UA=1|C)=\int_{l}\;P(UA=1|L)\cdot P(L|C)dL$$

Similarly, P(L|C) also cannot be measured at the time of decision-making; therefore, we must estimate this latent distribution using a variational autoencoder (VAE)^15^. We design a VAE that consists of a single hidden layer of size 128 that projects to the latent space P(L|C). Using this method, we can estimate P(L|C) at the time of decision-making, using only C. We can subsequently draw samples from P(L|C) to marginalize over L to obtain a final UA prediction P(UA=1|C).

The training objectives in a VAE are to learn both a latent space P(L|C) and a faithful reconstruction of the input data C. Continuous and binary variables were modeled separately as Gaussian and Bernoulli distributions, respectively. To accomplish this, three losses are used: Reconstruction, Kullback-Leibler (KL), and Evidence Lower Bound (ELBO) loss. Reconstruction loss is a numerical value that quantifies how well a generative model, such as a VAE, can reproduce the input by measuring the discrepancy between the original input and its reconstructed version. This incentivizes the model to preserve relevant information about the input. KL loss measures the distance between the latent distribution and the prior distributions. This incentivizes the model to learn a mapping of the input space to a smooth latent distribution. ELBO loss serves as an objective that combines both goals into a single training target. This is equivalent to simultaneously optimizing reconstruction and KL loss.

The reconstruction loss is calculated as the sum of two traditional supervised ML loss functions (mean squared error for continuous input variables and binary cross-entropy for binary input variables) that measure the error between our predicted distribution and the known training distribution L. Kullback-Leibler (KL) divergence is used to calculate the KL loss for the learned distribution to the respective Gaussian and Bernoulli distributions. The Evidence Lower Bound (ELBO) loss is used to optimize the VAE and is calculated as the sum of the reconstruction and KL losses. The VAE loss values on the training and test datasets are given in Table 3.

To estimate the probability, P(UA=1|C), we drew 1,000 samples from the VAE decoder latent distribution P(L|C). These samples were included in the input data for the LR model, along with clinical variables C, for UA prediction, and the resulting probabilities were averaged to obtain a final estimate marginalized over L. This Monte Carlo approach approximates the expected probability P(UA=1|C) under the learned latent distribution.

Once UA results are derived, the subsequent chance nodes labeled “UTI/No UTI” represent the probability of UTI before antibiotic initiation, given the predicted UA results. The probabilities represented by these chance nodes correspond to the PPV and NPV for UA=1 and UA=0, respectively. Individualized PPV and NPV were derived using:

$$P(UTI=1|UA=1,C)=\frac{P(UA\;=\;1|UTI\;=\;1,C)\;\cdot \;P(UTI\;=\;1|C)}{P(UA\;=\;1|C)}$$

$$P(UTI=0|UA=0,C)=\frac{P(UA\;=\;0|UTI\;=\;0,C)\;\cdot \;P(UTI\;=\;0|C)}{P(UA\;=\;0|C)}$$

where P(UA=1∣UTI=1,C) and P(UA=0∣UTI=0,C) are estimated using the UA model training averages, and P(UTI∣C) is the prior probability of UTI, given by UTICalc predictions.

If antibiotics are initiated, there is another chance node representing the probability of antibiotic resistance, yielding the final probability that the patient will have a persistent UTI after treatment. It is assumed that, in all cases, cephalosporins are prescribed and have a constant resistance rate of 0.1^16^. Lastly, we assumed that no antibiotics would be given if the risk of UTI was too low to warrant catheterization. It is worth noting that, in practice, clinicians may opt to initiate antibiotics without any testing; however, we excluded this option from the tree. This exclusion is due to the marginal gain in information from catheterization relative to its cost, which means that all utility functions in our tree always prefer to prescribe antibiotics without testing. Removing this branch results in a viable utility function and a tree that more closely resembles a standard gamble from decision theory^17^.

### Costs and Utilities

The EU was calculated as:

$$EU(\pi )=\sum_{i=1}^{n}\;P\left(o_{i}|\pi \right)\cdot U\left(o_{i}\right)$$

where π is a clinical decision, $o_{i}$ is a possible outcome, $P\left(o_{i}|\pi \right)$ is a model’s predicted probability of outcome $o_{i}$ after clinical decision π is taken, and $U\left(o_{i}\right)$ is the utility of outcome $o_{i}$. Predicted probabilities must be well-calibrated to accurately estimate expected utility. The optimal decision policy, π* maximizes EU:

$$\pi *=arg\underset{\pi }{max}\sum_{i=1}^{n}\;P\left(o_{i}|\pi \right)\cdot U\left(o_{i}\right)$$

The utility function used for the decision tree is designed as a function of the patient’s monetary cost. The costs are represented in Table 5. $E_{\left\{3,4\right\}}$ refers to a Level 3 or Level 4 visit to the emergency department. Patients who are catheterized are billed for a Level 4 visit, and other patients are billed for a Level 3 visit. Assumed costs for these visits are taken from Nationwide Children’s Hospital Prices, which specify the average Level 3 visit at $900 and the average Level 4 visit at $1300^18^.

For the cost of catheterization, T, we use the Centers for Medicare & Medicaid Services (CMS) physician fee schedule with HCPCS code 51701 (insert bladder catheter) at MAC Locality 1250299 (Rest of Pennsylvania), which gives a facility price of $23.67 for 2025^19^. For the cost of cephalosporin, , there is no straightforward source for what is billed to the patient since this is usually not charged separately for inpatient billing, according to CMS^20^. For purposes of this study, patients are expected to be billed approximately $4.

Lastly, we do not model the plausible case in which a patient is treated recursively with the tree through revisits. For simplicity, we assume that if a patient’s UTI is not resolved after one round of treatment, they will return for a Level 3 visit and be prescribed antibiotics. This assumption is reflected in the costs, where patients either resist the first round of antibiotics or have a persisting UTI after no treatment. Using these values, we define the ${Cost}_{\text{linear}}$ function, which represents the linear disutility of cost to a particular outcome. We further apply a quadratic transformation with min-max normalization to emphasize the accelerating burden on the patient as healthcare expenditure increases ^21^. To obtain a utility function for maximization, we take one minus the normalized cost. The quadratic transformation, min-max normalization, and utility functions are the following:

$${Cost}_{quad}\;={\left({Cost}_{\mathrm{linear}}-k\right)}^{2}$$

$${Cost}_{\mathrm{norm}}=\frac{{Cost}_{quad}\;-\;min\left({Cost}_{quad}\right)}{max\left({Cost}_{quad}\right)\;-\;min\left({Cost}_{quad}\right)}$$

$$\mathrm{Utility}\;=1\;-\;{Cost}_{norm}$$

**Figure 3.**
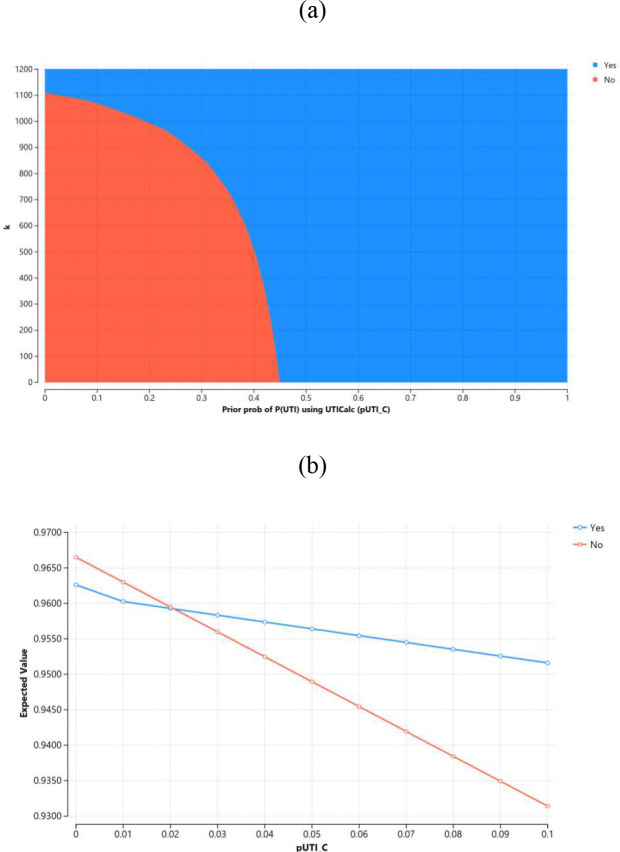
Sensitivity analysis of the UTI decision tree. Blue indicates decisions to catheterize, and red indicates decisions to not catheterize. (a) 2-way sensitivity analysis of cost function parameter k and P(UTI) informed by UTICalc. The blue and red regions meet at the indecision boundary. (b) 1-way sensitivity analysis using *k* = 1117.5 to show an indecision point at a prediction of 2% from UTICalc.

We find parameter k for ${Cost}_{\mathrm{quad}}$ using a 2-way sensitivity analysis on the tree using k and P(UTI) given by UTICalc; see Figure 3a. The cutoff value for UTICalc to classify a patient is 2%^14^, so this should be reflected in the decision tree’s decision boundary. A value of 1117.50 was determined to be the value for k which resulted in indecision at 2% probability (see Figure 3b).

### Fairness Analysis

To establish a baseline for prediction-based fairness, we evaluated equal opportunity across racial groups for both UTICalc V1 and V3. Equal opportunity is a fairness metric measuring parity in the true positive rate (TPR) across race. Formally, equal opportunity difference is defined as:

$$EOP=P(\overset{ˆ}{Y}=1|Y=1,A=1)-P(\overset{ˆ}{Y}=1|Y=1,A=0)$$

where Y and $\overset{ˆ}{Y}$ represent true and predicted outcome, respectively, and A represents a binary sensitive attribute. Calculations of all metrics are done on the test set. In the context of UTICalc, equal opportunity requires that children with a true UTI have an equal probability of receiving a high-risk prediction regardless of race. Disparities in equal opportunity indicate that the model is more sensitive to true cases in some racial groups than others, which may result in differential rates of diagnosis and treatment. Equal opportunity was selected as the prediction-based fairness metric because it directly reflects the clinical consequence of the screening test: that a child with a UTI should have an equal chance of being identified as high-risk regardless of race.

No standard fairness metrics are available for a decision-theoretic approach. We observe the number of decisions that change within each racial group by varying the UTICalc version. We also opt to measure disparities in EU and expected calibration error (ECE)^22^.

The difference in ECE is to observe a significant difference in the quality of calibration, a key assumption of decision theory^11^, for each racial group. These fairness metrics for EU parity and ECE parity are defined as:

$$\mathrm{EU}\;\mathrm{Parity}=E\left[U\left(X_{nb}\right)\right]-E\left[U\left(X_{b}\right)\right]$$

$$\mathrm{ECE}\;\mathrm{Parity}=\sum_{m=1}^{M}\;\frac{\left|B_{m}^{b}\right|}{n_{b}}\left|acc\left(B_{m}^{b}\right)-conf\left(B_{m}^{b}\right)\right|-\sum_{m=1}^{M}\;\frac{\left|B_{m}^{nb}\right|}{n_{nb}}\left|acc\left(B_{m}^{nb}\right)-conf\left(B_{m}^{nb}\right)\right|$$

where $X_{\left\{b,nb\right\}}$ indicates the racial groups of predictors $X,\;B^{\left\{b,nb\right\}}$ indicates binning of predicted probabilities using a given racial group, and $n_{\left\{b,nb\right\}}$ indicates the number of samples from the racial group. Terms $acc\left(B_{m}^{b}\right)$ and $conf\left(B_{m}^{b}\right)$ indicate the fraction of predictions in bin $B_{m}^{b}$ that were correct (i.e., the accuracy within the bin), and the average predicted probability in bin $B_{m}^{b}$. Results for ECE and ECE parity are included in the supplementary materials.

A significant difference in ECE across UTICalc versions or in ECE parity would indicate differential reliability of EU estimates across models or racial groups, respectively. Significant differences in EU or EU parity would indicate that decision-making for UTICalc V1 leads to suboptimal or unfair health outcomes for black patients relative to UTICalc V3. We test for both metrics for differences overall and within each group across versions, revealing if there are significant changes from UTICalc V1 to UTICalc V3. We use a non-parametric approach via bootstrap resampling (with 1,000 resamples) on the test data to construct 95% confidence intervals for each metric in a two-tailed test. Since a primary assumption of decision trees in utility theory is well-calibrated probabilities, we repeat these tests for recalibrated versions of UTICalc using simple histogram binning to post-process predicted probabilities^22^. Additionally, statistical testing is extended to individual EU by generating confidence intervals on individuals’ EU using a similar bootstrapping approach. We inspect how decisions change for individuals given each model and compare to changes in EU.

### Statistical Analysis

Categorical variables are presented as frequency (percentage). Differences in fairness metrics were assessed using a nonparametric bootstrap hypothesis test, in which data were resampled with replacement and the differences in metrics between models were recomputed. The procedure was repeated 1,000 times, each time with a sample size of 100, to construct an empirical null distribution. The 95% confidence interval was constructed using the corresponding quantiles from the bootstrap replicates. All calculations were performed using Python (v3.9.19). Decision tree design, expected utility calculations, and sensitivity analyses were performed using TreeAge Pro (v2024 R2).

## Supplementary Material

Supplementary Files

This is a list of supplementary files associated with this preprint. Click to download.

• Supplementaryinformationsubmission.docx

## Figures and Tables

**Table 1.** Catheterization rates and equal opportunity values of UTICalc clinical models V1 and V3. The values were computed using bootstrap samples from the test dataset. The p-values are the probabilities of observing a difference as extreme or more extreme than the observed difference in the test dataset, assuming the true difference is zero.

Metric	V1	V3	Difference	p-value

Catheterization Rate	0.5856 ± 0.0950	0.5616 ± 0.0951	−0.024 ± 0.095	0.6490
Equal Opportunity	0.4383 ± 0.336	0.1414 ± 0.1909	0.2969 ± 0.1485	<0.0001

**Table 2.** Expected utility (EU) and EU parity values of UTICalc V1 and V3 evaluated on the test dataset. The values were computed using bootstrap samples from the test dataset. The p-values are the probabilities of observing a difference as extreme or more extreme than the observed difference in the test dataset, assuming the true difference is zero.

Metric	UTICalc V1	UTICalc V3	Difference	p-value

EU	0.9504 ± 0.0042	0.9499 ± 0.0040	−0.0005 ± 0.0013	0.5360
EU parity	−0.0151 ± 0.0066	−0.0068 ± 0.0070	0.0083 ± 0.0027	0.4370

**Table 3.** Values of VAE evaluation metrics for training and test datasets. A lower ELBO value indicates better model performance.

Dataset	Reconstruction Loss	KL Divergence	ELBO (Loss)

Training	1345.0499	6.4182	1351.4681
Test	656.7698	5.8485	662.6183

**Table 4.** Description of probabilistic models in the decision tree.

Model	Probability Term	Description
UTI Present	P(UTI=1∣C)	An LR model that predicts the probability of UTI present from clinical information *C*.^1^
UA Positive	P(UA=1∣C)	An LR model that predicts the probability of a positive UA test from clinical information and urine analysis results. Since UA test results are unavailable at the time of decision-making, this probability is based on clinical information *C*.
PPV of UA	P(UTI=1∣UA=1,C)	Computed as: $\frac{P(UA\;=\;1|UTI\;=\;1,\;C)\;\cdot \;P(UTI\;=\;1|C)}{P(UA\;=\;1|C)}$ (Sensitivity of UA * UTI Present / UA Positive)
NPV of UA	P(UTI=0∣UA=0,C)	Computed as: $\frac{P(UA\;=\;0|UTI\;=\;0,\;C)\;\cdot \;P(UTI\;=\;0|C)}{P(UA\;=\;0|C)}$ (Specificity of UA * (1 - UTI Present) / (1 - UA Positive)

1 *C* describes different EHR data across model versions; V1: age (<12 months vs. >=12 months), sex (female or uncircumcised male vs. circumcised male), race (Black vs. nonblack), fever magnitude (>39°C vs. =<39°C), and alternate fever source (absence of another source of fever vs. presence), V3: age, sex, fever magnitude, alternate fever source, duration of fever (>=48 hours vs. <48 hours), and history of UTI (yes vs. no).

**Table 5.** *Cost function for values of catheterization, antibiotics, UTI, and antibiotic resistance. E*_{3,4}_ *represents the cost of a “Level 3” or “Level 4” emergency department visit. A represents the cost ofprescribing antibiotics. T represents the cost of catheterization.*

Catheterization	Antibiotics	UTI	Resistant	Cost*_linear_*

No	No	Absent	No	*E*_3_
No	No	Present	No	2*E*_3_ + *A*
Yes	No	Absent	No	*E*_4_ + *T*
Yes	No	Present	No	*E*_4_ + *T* + *E*_3_ + *A*
Yes	Yes	Absent	No	*E*_4_ + *T* + *A*
Yes	Yes	Present	No	*E*_4_ + *T* + *A*
Yes	Yes	Present	Yes	*E*_4_ + *T* + *E*_3_ + 2*A*

## Data Availability

The data used in this study are not publicly available because they contain protected health information and were collected under an Institutional Review Board (IRB) protocol at the University of Pittsburgh. Researchers interested in accessing the data may contact the corresponding author to inquire about potential collaboration or data use agreements, subject to IRB approval.
